# Use of Oral Vitamin-D Glass ampoule and tablet: Experience of patients and physicians

**DOI:** 10.12669/pjms.332.12454

**Published:** 2017

**Authors:** Syed Umair Maroof, Faizan Shaukat, Junaid Aslam, Masood Jawaid

**Affiliations:** 1 Syed Umair Maroof, B-Pharm, MBA PharmEvo [Pvt] Limited, Karachi, Pakistan; 2 Dr. Faizan Shaukat, MBBS Senior Executive, Medical Affairs, PharmEvo [Pvt] Limited, Karachi, Pakistan; 3 Junaid Aslam, MBA PharmEvo [Pvt] Limited, Karachi, Pakistan; 4 Dr. Masood Jawaid, MBBS, MCPS, MRCS (Glasg), FCPS, MHPE Consultant General Surgeon, Darul Sehat Hospital and Director Medical Affairs, PharmEvo [Pvt] Limited, Karachi, Pakistan

**Keywords:** Vitamin D, Hazard, Ampoules, Tablets, Oral medication, Compliance

## Abstract

**Objective:**

To ascertain patients and physician views regarding hazard and compliance of oral liquid Vitamin D glass ampoule and tablets.

**Methods:**

This cross sectional survey was conducted from November 1^st^ 2016 to 15^th^ December 2016. Patients who were prescribed Vitamin D glass ampoule from oral route in last three months were included along with physicians who routinely prescribe vitamin D after taking informed consent. The participants were asked about injuries related to the use of glass ampoule, ease of using this from, after taste preference of tablet or injectable form as well as demography. Data was analysed with SPSS version 24.0.

**Results:**

Total 182 patients were included in the study with mean ± SD age of 39.4 ± 12.4 years. Majority of patients, 80.2% (142) said they prefer oral tablet in preference to injectable ampule in oral form if given choice while prescribing Vitamin D. Moreover 66.7% (64) doctors prefer to prescribe tablet form of Vitamin D instead of injection as oral form for vitamin D deficiency among their patients. One third of patients, 33% (n=59) sustained injury while breaking the ampule which included minor self-controlled bleeding by glass particles in 50% (n=35). Less than half of doctors 46.9% (n=45) said they taught their patients about usage of injectable Vitamin D ampules.

**Conclusion:**

Majority of patients prefer Vitamin D tablet instead of Oral liquid in glass ampoule if they got the choice among two. The results of this study provide important implications for our doctors about patients concern of hazard, after taste and compliance with orally administered Vitamin D glass ampoules.

## INTRODUCTION

Vitamin D deficiency is pandemic and is present in more than one billion people all over the world.^1^ The occurrence of vitamin D deficiency/insufficiency is high not only in countries where sunlight is less but as well in countries near equator because of their traditional and religious clothing habits and life style.^2^ Most of the persons with vitamin D deficiency/insufficiency are asymptomatic and hence making diagnosis of Vitamin D deficiency is very challenging. Vitamin 25(OH) D levels less than 20 ng/ml are taken as cut off for vitamin D deficiency.^3^ Hypovitaminosis D has been associated with various diseases including hypertension, cardiovascular diseases (CVD), type-1 and type-2 diabetes mellitus (DM), obesity, dyslipidemia, congestive heart failure, multiple sclerosis, Parkinson disease, rheumatoid arthritis, cognitive impairment, several types of cancers such as prostate, breast and colon cancers, viral and bacterial infections, chronic obstructive pulmonary diseases, autoimmune diseases, dementia and complications of pregnancy.^4^-^6^ Pakistani population is also very deficient in Vitamin D. It is reported that up to 91.50% of some areas were found to have Vitamin-D deficiency in Karachi.^7^

There are various forms of Vitamin D supplements available ranging from Oral chewable table to Liquid Vitamin D in glass ampule. Opening ampoules may expose professionals to percutaneous injuries. These lesions can represent an important biological risk as it can lead to contamination with bacteria and viruses. Ampoule packaging may also represent a potential source of microbial infection for patients. Several measures are required for risk prevention, such as the use of gloves; gauze, ampoule openers, as well as disinfection of ampoules with 70% alcohol before opening are available to professionals handling such vessels.^8^,^9^

Apart from tablets many healthcare physicians prescribe injectable glass ampules of Vitamin D which apart from parentally can be given orally. Author’s organization recently launched oral form of Vitamin D and they were interested in evidence about different Health Care Physicians (HCP) practices and perception for different oral modality of Vitamin D. Despite the literature showing concerns for this practice^8^, we were unable to find any evidence about the experiences, concern and preferences of both practices among patients as well as health care physicians. This survey was designed to find the common experiences about orally administered glass ampoules and oral tablets of Vitamin D in our population and the general perception regarding its safety and hazards.

## METHODS

This cross sectional survey was conducted from November 1^st^ 2016 to 15^th^ December 2016 in different outpatient department of different hospitals in Karachi, Lahore and Islamabad. Patients who were prescribed Vitamin D glass ampoule from oral route in last three months were included along with physicians who routinely prescribe vitamin D after taking informed consent. The participants were asked about injuries related to the use of glass ampoule, ease of using this form, after taste preference of tablet or injectable form as well as demography. Response rate for patients was 60.2% while for doctors was 80%. Data was analyzed with SPSS version 24.0.

## RESULTS

Total of 182 patients and 96 doctors who fulfilled the inclusion criteria were included in the study. Out of 182 patients, 61.5 (%) were male while 38.5 (%) were female with mean ± SD age of 39.4 ± 12.4 years. All patients were prescribed vitamin D in oral form from injectable glass ampules. More than half of patients, 52.8% (95) said that no consent was taken from them while prescribing this medicine while 39.7% (71) were formally taught how to break and use the ampule in oral form. One third of patients, 33% (59) sustained injury while breaking the ampule which included minor self-controlled bleeding by glass particles in 50% (35) followed by minor laceration in 27.1% (19) of patients. Overall 48% (86) of patients experienced spillage of drug from sometime to frequently in last one month. Most of the patients, 80.2% (142) said they prefer oral tablet in preference to injectable ampule form if given choice while prescribing Vitamin D. All results are shown in Table-I.

**Table-I:** Patients experiences of oral vitamin D injection usage (n=182).

*Questions*	*N (%)*

	Yes
Has your healthcare provider taken your consent before prescribing injection form of Vitamin D from oral route?	85 (47.5)
Were you taught by health care professional how to properly break glass ampule?	71 (39.7)
Did you break glass ampule yourself?	87 (48.6)
Did you feel comfortable breaking the glass ampule?	59 (32.6)
Did you injure yourself while breaking glass ampoule?	59 (33.0)
***What was the extent of Injury***	
Laceration	19 (27.1)
Self-controlled bleeding	35 (50.0)
Bleeding that required bandaging	4 (5.7)
Others	12 (17.1)
***How often you spill Liquid Vitamin D out while breaking glass ampule?***	
Frequently	15 (8.4)
Often	14 (7.8)
Sometimes	57 (31.8)
Rarely	40 (22.3)
Never	52 (29.1)
Have you ever found a broken glass piece in your medicine while taking it?	30 (16.6)
Is there any bad taste after taking Liquid Vitamin D injection ampule?	89 (49.4)
***If given preference which form of medication you will choose?***	
Oral tablet	142 (80.2)
Liquid in glass ampoule	35 (19.8)

Total 96 doctors who prescribe Vitamin D were included in the study. Most of them, 66.7% (64) prefer to prescribe table form of Vitamin D instead of injection as oral form for vitamin D deficiency among their patients. Less than half i.e. 46.9% (45) said they taught their patients about usage of injectable Vitamin D ampules. On inquiring which form of prescription results in better compliance they said oral tablet form 74% (71). Only 19.8% (19) were of the opinion that injectable form given in oral form has better bioavailability while 49% (47) think there is no difference (Table-II).

**Table-II:** Health care physicians perspective of Vitamin D prescription (n=96).

*Questions*	*Yes*

	N (%)
What do you normally prefer for your patient in Vitamin D deficiency?	
Tablet	64 (66.7)
Oral Liquid Injection form	32 (33.3)
Do you normally demonstrate or teach them how to break glass ampoule?	45 (46.9)
Have any patient requested you to switch from Liquid vitamin D to oral Vitamin D	46 (47.9)
Have any of your patient suffered some injury while breaking glass ampoule?	42 (43.8)
Patients have better compliance with which Form, Injection or Tablet?	
Chewable tablet	71 (74)
Oral liquid	25 (26)
Which form of iron preparation has better bioavailability?	
Chewable tablet	30 (31.3)
Oral Liquid	19 (19.8)
Both	47 (49)

## DISCUSSION

According to this study, 52.5% doctors do not take consent from patients before prescribing injection form of Vitamin D. Almost 80% patients will chose oral tablet if given the choice. Only 46.9% physicians demonstrate how to break an ampoule to their patients. The study was first of its kind in term of documenting patient’s and doctor experiences about the hazard, compliance, risk of two most common form of Vitamin D; oral glass ampoules and oral tablets. Varieties of options are available for individual vitamin D supplements, including capsules, chewable tablets, liquids, and drop.^1^ Dosing is either 25,000 IU every fortnight for 8 weeks (total dose 100,000 IU), 25,000 IU every week for 6 weeks (total dose 150,000 IU), or 25 000 IU every week for 8 weeks (total dose 200,000 IU).^10^ There are over 100 different labels available for Vitamin D in Pakistan. On average, Oral chewable are slightly expensive than oral liquid form.

Opening ampoules is a particularly high-risk event. Sharps injuries (SIs) were caused by opening an ampoule.^11^ Most frequently reported circumstances of sharps injuries were opening of ampoules and vials, 12 which may result in compliance issue with Vitamin D ampoules. In our study 33% patients had some cut injury while opening the ampoule, of which 5.7% required bandaging. Another risk associated with glass ampoules is particle contamination.^13^

Recent estimates for noncompliance vary from study to study and ranges from 62 to 84 percent using electronic monitoring.^14^,^15^ Hence, it is very important for physician to eliminate all possible factors that might influence patient compliance. In our study only 32.6% (n=52) felt comfortable breaking the glass ampoules and rest relied on other to break the ampoule. Patients who are active participants in a shared decision-making process have a better knowledge of treatment options and more realistic perceptions of likely treatment effects. The resulting treatment choices are more likely to concur with their preferences and attitudes to risk.^16^ Only 47.5% (n=85) patients said that their doctor took consent for giving them Vitamin D in oral liquid ampoule. Physicians are inclined to prescribe oral liquid form as they believe its bioavailability is higher; however there is no clinical data available for comparison of bioavailability of both forms. It is well established that actively engaged patients are also more likely to adhere to treatment recommendations.^16^ In our study, only 39.7% (n=71) patients said that their physician demonstrated them how to break a glass ampoule. About 46.9% (n=45) doctors said that they teach their patients how to break ampoule. Few pharmacies of hospitals also train patient how to break a glass ampoule but the practice is very rare in Pakistan health care system. Oral liquid medications with poor palatability may lead to non-compliance.^17^ In our study 49.4% of patients experience bad taste after taking vitamin D from glass ampoule. About 74% physicians believe that compliance of patient on vitamin D tablets is better than patients on oral liquid form.

It is empirical that doctors should take consent from patients before prescribing any form of vitamin D and take into consideration the hazard related to oral vitamin D in glass ampules. Proper training to break ampoule must be given to patients to combat their fear and reluctance to break glass ampoule. However, it is better if physicians prescribe tablets of Vitamin D if available.

### Limitations of the Study

Despite providing some useful information, this study has certain limitations. Firstly only a small number of patients were enrolled in the study. There is a need for much larger multi-centre study to confirm our findings.

### Authors’ Contributions

***SUM:*** Concept and design of the study.

***FS:*** Data collection, data entry and analysis and drafted the manuscript.

***MJ:*** Worked on concept and design of study, analyzed results and was involved in preparing the manuscript.

***JA:*** Collected data and review of literature.

All authors have read and approved the final version of the manuscript to be published
